# Glaucocalyxin A Inhibits the Malignant Progression of Epithelial Ovarian Cancer by Affecting the MicroRNA-374b-5p/HMGB3/Wnt-β-Catenin Pathway Axis

**DOI:** 10.3389/fonc.2022.955830

**Published:** 2022-07-14

**Authors:** Feng Chen, Fang Sun, Xia Liu, Jing Shao, Bei Zhang

**Affiliations:** ^1^ Department of Gynecology and Obstetrics, Xuzhou Central Hospital Affiliated to Nanjing University of Chinese Medicine, Xuzhou, China; ^2^ Department of Gynecology and Obstetrics, Xuzhou Central Hospital, Xuzhou, China; ^3^ Department of Pathology, Xuzhou Central Hospital, Xuzhou, China; ^4^ Department of Clinical Laboratory, Xuzhou Central Hospital, Xuzhou, China

**Keywords:** glaucocalyxin A, high-mobility group box 3, epithelial ovarian cancer, microRNA-374b-5p, Wnt-β-catenin pathway

## Abstract

**Objective:**

Glaucocalyxin A (GLA) is an ent-kaurene diterpenoid from *Rabdosia japonica* var possessing anti-tumor activity. This study aimed to investigate effects of GLA on epithelial ovarian cancer (EOC) and elucidate underlying mechanisms.

**Methods:**

The expression of HMGB3 in EOC tissues was analyzed by GEPIA and immunohistochemistry. Cell proliferation was determined using CCK-8 and colony formation assays. Cell invasion, migration, and apoptosis were detected using Transwell, wound healing, and flow cytometry assays, respectively. Interactions between HMGB3 and miRNAs were predicted using ENCORI and validated using a dual-luciferase assay. mRNA expression levels of HMGB3 and miRNAs were measured using qPCR. Protein expression levels of HMGB3, E-cadherin, N-cadherin, Wnt3a,β-catenin, Bcl-2, and Bax were measured by western blotting. A tumor xenograft model was established to validate the efficacy and mechanism of GLA *in vivo*.

**Results:**

HMGB3 was upregulated in EOC tissues and cells. GLA dose-dependently inhibited EOC cell proliferation and epithelial-mesenchymal transition (EMT). HMGB3 overexpression promoted proliferation, invasion, migration, and EMT, and suppressed the apoptosis of EOC cells. In addition, miR-374b-5p was targeted by HMGB3, and its overexpression hindered malignant characteristics of EOC cells. HMGB3 overexpression weakened antitumor effects of GLA and miR-374b-5p in EOC cells. Moreover, the Wnt-β-catenin pathway was inhibited by the GLA-mediated miR-374b-5p/HMGB3 axis. *In vivo* experiments showed that GLA inhibited EOC tumor growth, meanwhile, upregulated the miR-374b-5p level and downregulated the expression of HMGB3, Wnt3a, and β-catenin in tumor tissues.

**Conclusions:**

GLA suppressed the malignant progression of EOC by regulating the miR-374b-5p/HMGB3/Wnt-β-catenin pathway axis.

## Introduction

Epithelial ovarian cancer (EOC) results in the most deaths compared with those of other female reproductive cancers worldwide, with 152,000 deaths and 239,000 new cases occurring annually (1). Routine therapeutic strategies for EOC mainly include cytoreductive surgery and combination platinum-taxane chemotherapy (2). Although the treatment of EOC has greatly advanced in recent years, their therapeutic effects remains limited, and the survival rate remains unsatisfactory (3). Therefore, developing more efficacious drugs for EOC treatment is urgently needed.

Glaucocalyxin A (GLA) is a bioactive ent-kaurene diterpenoid extracted from the Chinese herb *Rabdosia japonica* var, with various beneficial biological activities, such as anti-cancer, anti-inflammatory, and immune regulation effects (4). GLA has been shown to exert favorable effects against various cancers, such as gastric, bladder, liver, and breast cancer (5–8). For instance, GLA exerts inhibitory effects on the epithelial-mesenchymal transition (EMT) and invasion of gastric cancer cells (8). In human bladder and breast cancer cells, GLA induces apoptosis and arrests cell cycle in the G2/M phase (5, 6). GLA exerts antiproliferative and apoptotic effects in liver cancer cells (7). However, to our knowledge, no study has focused on therapeutic effects of GLA in EOC.

High-mobility group box 3 (HMGB3) is a critical regulator of cell proliferation and apoptosis in multiple cancers, including breast cancer, lung cancer, and prostate cancer (9–12). Mukherjee et al. have also showed that HMGB3 is upregulated in ovarian cancer cells, and its depletion promotes apoptosis of ovarian cancer cells (11). Nevertheless, the underlying mechanisms of HMGB3 in EOC have not yet been fully elucidated. Wnt-β-catenin pathway, which regulates cell proliferation, differentiation, and invasion, is a potential downstream pathway of HMGB3 (13, 14). HMGB3 participates in modulating cancer development by affecting the Wnt-β-catenin pathway (15–20). For instance, HMGB3 serves as an oncoprotein that facilitates the proliferation of cervical cancer cells by activating the Wnt-β-catenin pathway (16, 19). Silencing HMGB3 slows cell proliferation and invasion in non-small cell lung cancer by inhibiting the Wnt-β-catenin pathway (17). HMGB3 also exerts an oncogenic role in colorectal cancer *via* activating the Wnt-β-catenin pathway (15). Therefore, we hypothesize that GLA protects against EOC by regulating HMGB3 and its downstream Wnt-β-catenin pathway.

MicroRNAs (miRNAs), a subtype of short non-coding RNAs, are pivotal regulators of biological processes in human cancers (21). Several miRNAs are closely associated with tumor progression by regulating the HMGB3/Wnt-β-catenin pathway axis (18, 20, 22). Sun et al. have found that hypermethylation of miR-216a promotes malignant proliferation and metastasis of esophageal cancer by upregulating HMGB3 and activating the Wnt-β-catenin pathway (20). Xie et al. have reported that miR-532-5p suppresses the proliferation and invasion of bladder cancer cells *via* targeting HMGB3 and hindering the Wnt-β-catenin pathway (22). However, it is still unclear which miRNAs play a key role in the therapeutic effect of GLA against EOC by regulating the HMGB3/Wnt-β-catenin pathway axis.

In this study, antitumor effects of GLA on EOC were studied *in vitro*. Moreover, the underlying mechanisms of GLA against EOC involving the miR-374b-5p/HMGB3/Wnt-β-catenin pathway axis were elucidated. Here, we present a novel and effective drug for EOC treatment and provide insights on the potential mechanism of action of GLA against EOC.

## Methods

### Clinical Sample Collection

Human normal and EOC tissues were obtained from patients with EOC during surgery, followed by immediately storing in liquid nitrogen and used for immunohistochemistry to detect HMGB3 expression. Oral informed consents were obtained from patients for this study. This study was approved by the Ethics Committee of Xuzhou Central Hospital (XZXY-LK-20210520-002).

### Cell Culture and Treatment

Human EOC cell lines SKOV3 and OVCAR3 and normal human ovarian epithelial cell line IOSE80 were obtained from the Shanghai Cell Bank (Chinese Academy of Sciences, China). All cells were cultured in RPMI-1640 medium (Gibco, CA, USA) supplemented with 10% fetal bovine serum (FBS) and 1% penicillin/streptomycin (Gibco, CA, USA) at 37 °C with 5% CO_2_. SKOV3 and IOSE80 cells were divided into six groups to determine the optimal GLA treatment concentration: control group and five GLA groups (1, 2, 4, 8, and 16 μmol/L GLA).

### Cell Transfection

Lentivirus packaged shRNA negative control (sh-NC) and sh-HMGB3 were purchased from Sigma (MO, USA). Lentivirus negative control (lenti-NC) and lentivirus containing HMGB3 (lenti-HMGB3) were purchased from Addgene (Cambridge, MA, USA). MiR-374b-5p mimics and negative control mimics (NC mimics) were purchased from GenePharma (China). These agents were transfected with SKOV3 cells using Lipofectamine 3000 (Invitrogen) for 48 h.

### Cell Proliferation Assay

SKOV3 cells were cultured in 96-well plates (5 × 10^3^ cells/well) for 24, 48, or 72 h, followed by incubating with 10 µL the cell counting kit-8 (CCK-8; Beyotime, China) solution for 2 h at 37 °C. Absorbance at 450 nm was detected under a DR-200Bs microplate reader (Diatek, China). The relative half-maximal inhibitory concentration (IC50) value of GLA was determined 48 h after culturing according to a cell proliferation curve.

### Colony Formation Assay

SKOV3 cells were seeded in 6-well plates (200 cells/well) for seven days to allow colony formation. After washing twice with PBS, SKOV3 cells were immobilized with ethanol for 15 min and then stained with crystal violet (BASO, China) for 20 min. Subsequently, cells were photographed using a digital SLR camera (Nikon, Japan).

### Cell Invasion Assay

For cell invasion detection, 200 μL resuspended SKOV3 cells (1 × 10^5^ cells/mL) were plated into the upper chamber of a Matrigel-coated Transwell, and 600 μL RPMI-1640 medium with 20% FBS (Gibco, CA, USA) was added to the lower chamber. Following 24 h incubation, cells in the lower chamber were immobilized with methanol at 4 °C for 30 min, followed by staining with crystal violet for 20 min. After washing with PBS, cell images were captured under a microscope by randomly selecting five fields and were counted using ImageJ.

### Wound Healing Assay

SKOV3 cells were cultured overnight in six-well plates (5 × 10^5^ cells/well). A wound was created using 200 µL pipette tips for cells in each well, followed by culturing for 48 h. The healing distance was photographed before and after culturing, and the wound-healing rate was calculated.

### Cell Apoptosis Assay

SKOV3 cells (300 μL, 1 × 10^5^ cells/mL) were incubated with 5 µL annexin V-FITC for 15 min, and then with 10 µL propidium iodide for 10 min in the dark. Cell apoptosis was determined using a flow cytometer with Cell Quest software (BD Biosciences, NJ, USA).

### Bioinformatics Analysis

HMGB3 expression profiles in EOC tissues were analyzed using the Gene Expression Profiling Interactive Analysis (GEPIA, http://gepia.cancer-pku.cn/) database. Potential miRNAs targeting HMGB3 and the binding sites were predicted using the Encyclopedia of RNA Interactomes (ENCORI, http://starbase.sysu.edu.cn/index.php) (23).

### Dual-Luciferase Reporter Assay

Binding of miR-374b-5p to HMGB3 was validated using a dual-luciferase reporter assay. Wild-type (HMGB3-Wt) and mutant HMGB3 (HMGB3-Mut) were cloned into luciferase vectors (Beyotime). Recombinant HMGB3-Wt and HMGB3-Mut were co-transfected with miR-374b-5p mimics into SKOV3 cells. Luciferase activities of Firefly and Renilla were detected by the Dual-Luciferase Reporter Assay System (Promega, WI, USA) at 48 h post-transfection.

### *In Vivo* Xenograft Experiments

Animal experiments were approved by the Institutional Animal Care and Use Committee and local experimental ethics committee. Four-week-old female BALB/c nude mice (n = 6 per group) were obtained from HFK Bioscience Co. Ltd. (Beijing, China) and adaptively raised for three days, supplemented with adequate food and water. To establish a xenograft model, SKOV3 cells were transfected into mice *via* subcutaneous injection (1 × 10^6^ cells/injection site). The tumor-bearing mice were raised for one week and then were subcutaneously injected with low- (15 mg/kg) or high-dose (30 mg/kg) GLA. After 30 days, mice sacrificed by cervical dislocation. The diameter of tumors was measured using a vernier caliper every three days. Tumor volumes were calculated as follows: volume (mm^3^) = (width^2^ × length) × 1/2.

### Immunohistochemistry

EOC tissues were embedded in paraffin and then sectioned into 4 µm-thick slides for immunohistochemical staining. Before staining, tissue sections went through deparaffinating, rehydration, and antigen retrieval. Following this, sections were blocked for endogenous peroxidase by incubating with 3% H_2_O_2_ for 20 min. Subsequently, each section was incubated with 50 μL normal goat serum and subjected to incubation with anti-HMGB3 or anti-Ki-67 primary antibody (1:100; Abcam, UK) at 4 °C overnight. After washing thrice with phosphate-buffered saline (PBS; Solarbio, China), tissue slides were incubated with secondary antibody (1:50; MultiSciences, China) for 15 min and then with streptavidin-biotin-peroxidase solution for 15 min. Next, slides were stained with diaminobenzidine (DAB; Changdao, China) and hematoxylin (BASO, China) for three min. Subsequently, sections were photographed under a light microscope (Olympus).

### qPCR

Total RNA from EOC tissues and cells was isolated using TRIzol (Invitrogen, CA, USA). cDNA was prepared for qPCR by reverse transcription using the EasyScript^®^ First-Strand cDNA Synthesis SuperMix (TransGen, China). PCR was conducted using a SYBR Green PCR Master Kit (Lifeint, China) under an Mx3000P Real-Time PCR System (Stratagene, CA, USA) with a PCR reaction program of 95 °C for 3 min, 40 cycles of 95 °C for 12 s, and 62 °C for 40 s. Primers are listed in **Table 1**. GAPDH was utilized as an internal reference for HMGB3 and U6 for miRNAs. Relative expression was calculated using the 2^-ΔΔCt^ method.

**Table 1:** Primers used in qPCR.

Genes	Sequences of primers
HMGB3	Forward: 5′-GTC CGC TTA TGC CTT CT-3′ Reverse: 5′-CAT CGT CTT CCA CCT CT-3′
miR-374b-5p	Forward: 5′-CGG ATT AGG CAC TGT GAA TAC AAA G-3′ Reverse: 5′-TCT GCC AGG TAG AGT GGG AAA C-3′
miR-429	Forward: 5′-GCC GAT TAA TAC TGT CTG GTA A-3′ Reverse: 5′-CAG TGC AGG GTC CGA GGT-3′
miR-214-3p	Forward: 5′-ACA GCA GGC ACA GAC AGG CAG T-3′ Reverse: 5′-TGC CTG TCT GTG CCT GCT GTT T-3′
GAPDH	Forward: 5′-ACA ACT TTG GTA TCG TGG AAG G-3′ Reverse: 5′-GCC ATC ACG CCA CAG TTT C-3′
U6	Forward: 5′-GCT TCG GCA GCA CAT ATA CTA AAA T-3′ Reverse: 5′-CGC TTC ACG AAT TTG CGT GTC AT-3′

### Western Blot Analysis

Western blotting was carried out as previously described (24). The anti-rabbit primary antibodies used for western blotting included anti-E-cadherin, -N-cadherin, -HMGB3, -Wnt3a, -β-catenin, -Bcl-2, -Bax, and -GAPDH (1:1,000; Abcam, UK), and the secondary antibody was horseradish peroxidase-conjugated (1:500; MultiSciences, China). Protein bands were visualized using an ECL reagent kit (Thermo Fisher Scientific, CA, USA) and ChemiDoc™ imaging system (Bio-Rad, CA, USA). GAPDH was used as a reference.

### Statistical Analysis

All data are presented as the mean ± standard deviation. Comparisons between different groups were performed using one-way analysis of variance and Tukey’s test. Statistical analysis was performed using GraphPad Prism 8. P < 0.05 was statistical significance.

## Results

### HMGB3 Was Upregulated in EOC Tissues and Cells

**Figure 1:**
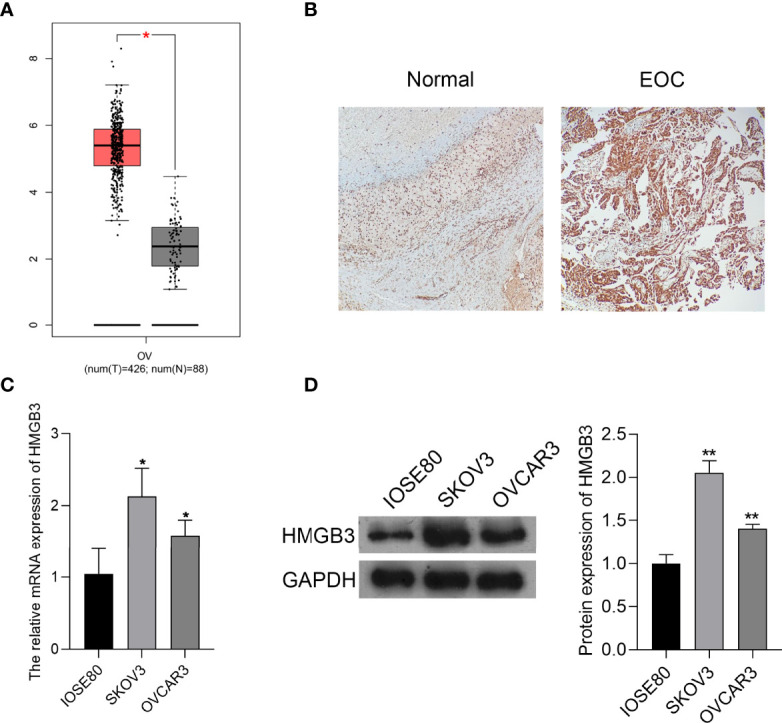
HMGB3 was highly expressed in epithelial ovarian cancer (EOC) tissues and cells. **(A)** The HMGB3 expression profile was analyzed in EOC tissues according to the data on the gene expression profiling interactive analysis (GEPIA, http://gepia.cancer-pku.cn/). ^*^ *P* < 0.05. **(B)** HMGB3 expression in human EOC tumor tissues and normal tissues was detected by immunohistochemistry (100× magnification). **(C)** The mRNA expression of HMGB3 in EOC cell lines (SKOV3 and OVCAR3) and the normal human ovarian epithelial cell line (IOSE80) was measured by qPCR. **(D)** Relative protein expression of HMGB3 in EOC cell lines (SKOV3 and OVCAR3) and the normal human ovarian epithelial cell line (IOSE80) was determined by western blotting. ^*^ *P* < 0.05 and ^**^ *P* < 0.01 vs. IOSE80.

HMGB3 is involved in the recurrence, progression, and drug resistance of various cancers (25). According to GEPIA data, the expression of HMGB3 was significantly higher in EOC tumor tissues than that in normal tissues (*P* < 0.05) (**Figure 1A**). Immunohistochemical staining also revealed that HMGB3 expression was higher in human EOC tissues than that in normal tissues (**Figure 1B**). Furthermore, the relative mRNA expression of HMGB3 was remarkably higher in the EOC cell lines (SKOV3 and OVCAR3) than that in the normal IOSE80 cells (*P* < 0.05). HMGB3 presented a relatively higher level in SKOV3 cells than that in OVCAR3 cells; therefore, SKOV3 cells were used for subsequent experiments (**Figures 1C, D**).

### GLA Inhibited the Metastasis and Proliferation of EOC Cells

**Figure 2:**
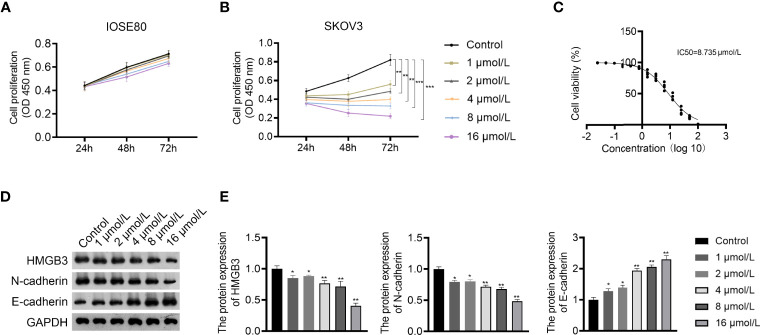
Glaucocalyxin A (GLA) dose-dependently inhibited proliferation, epithelial-mesenchymal transition (EMT), and HMGB3 expression in EOC cells. **(A, B)** The proliferation of IOSE80 and SKOV3 cells was measured by CCK-8 assay. **(C)** The IC50 value of GLA was determined by a proliferation curve at 48 h post-treatment. **(D, E)** The protein levels of HMGB3 and EMT-related biomarkers (E-cadherin and N-cadherin) in SKOV cells were detected by western blotting. EOC cells were treated with 1, 2, 4, 8, and 16 μmol/L GLA, respectively. *P <0.05 and **P <0.01 vs. control..

GLA is an ent-kaurene diterpenoid with antitumor activity and was used to treat EOC cells in this study. The CCK-8 assay showed that GLA dose-dependently suppressed the proliferation of SKOV3 cells (*P* < 0.01) (**Figures 2A, B**). The IC50 value of GLA was 8.735 µmol/L, which was used for subsequent experiments (**Figure 2C**). E-cadherin and N-cadherin are important players in the EMT process and are associated with cancer cell metastasis (26). Western blotting demonstrated that GLA administration dose-dependently upregulated E-cadherin expression and downregulated N-cadherin levels in SKOV3 cells compared to those in the control (*P* < 0.05) (**Figures 2D, E**). In addition, GLA dose-dependently decreased the expression of HMGB3 (*P* < 0.05) (**Figures 2D, E**).

### GLA Suppressed the Malignant Characteristics of EOC Cells *via* Regulating HMGB3

**Figure 3:**
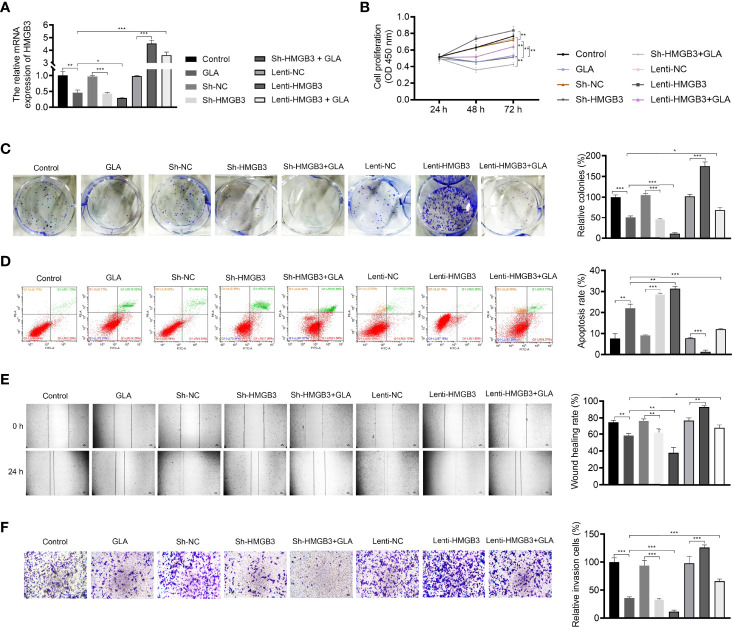
GLA inhibited the malignant characteristics of EOC cells by regulating HMGB3. **(A)** The relative mRNA expression of HMGB3 in SKOV3 cells was measured by qPCR. **(B)** The viability of SKOV3 cells was detected by CCK-8 assay. **(C)** The proliferation of SKOV3 cells was detected by colony formation assay. **(D)** The apoptosis of SKOV3 cells was detected by flow cytometry. **(E)** The migration of SKOV3 cells was detected by wound healing assay. **(F)** The invasion of SKOV3 cells was detected by the Transwell assay. Scale bar = 50 µm. SKOV3 cells were treated with GLA, sh-HMGB3/sh-NC, and/or lenti-HMGB3/lenti-NC. ^*^ *P* < 0.05, ^**^ *P* < 0.01, ^***^ *P* < 0.001.

**Figure 4:**
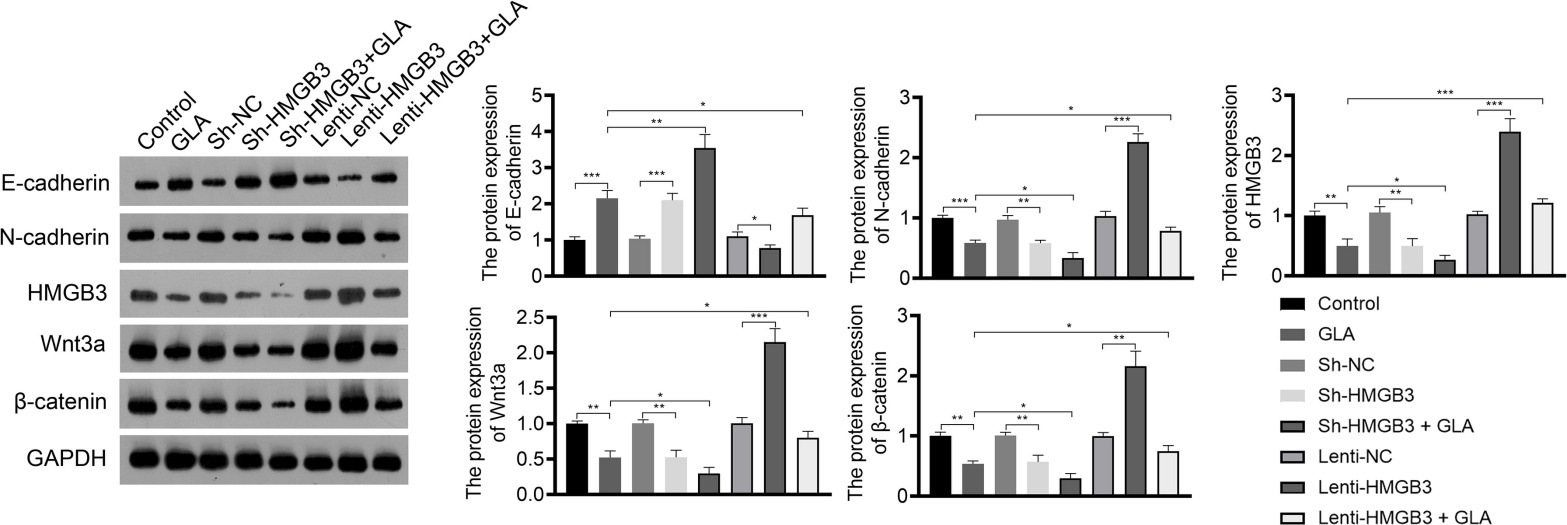
GLA inhibited the expression of EMT and Wnt-β-catenin pathway-related proteins by regulating HMGB3. The relative protein expression of E-cadherin, N-cadherin, HMGB3, Wnt3a, and β-catenin in SKOV3 cells was measured by western blotting. SKOV3 cells were treated with GLA, sh-HMGB3/sh-NC, and/or lenti-HMGB3/lenti-NC. ^*^ *P* < 0.05, ^**^ *P* < 0.01, ^***^ *P* < 0.001.

The mechanism of action of GLA, involving HMGB3, was analyzed in SKOV3 cells. **Figure 3A** presents the decreased and increased HMGB3 gene expression in cells transfected with sh-HMGB3 and lenti-HMGB3 when compared to that in NC cells, respectively (*P* < 0.001). GLA treatment downregulated the HMGB3 expression in cells transfected with sh-HMGB3 and lenti-HMGB3 (**Figure 3A**). HMGB3 knockdown significantly reduced the cell proliferation and relative colonies, and induced apoptosis of SKOV3 cells compared with those in sh-NC (*P* < 0.01) (**Figures 3B–D**). Meanwhile, western blotting showed that GLA inhibited the expression of Bcl-2 (an anti-apoptotic protein) and increased the Bax (a pro-apoptotic protein) level in SKOV3 cells, however, lenti-HMGB3 offset the effect of GLA (*P* < 0.01) (**Supplementary Figure 1**). The migration and invasion abilities of SKOV3 cells were significantly inhibited by HMGB3 knockdown (*P* < 0.01) (**Figures 3E, F**). EMT was also obviously decreased in SKOV3 cells after HMGB3 knockdown, as evidenced by the increased E-cadherin and decreased N-cadherin compared to those in sh-NC (*P* < 0.01) (**Figure 4**). HMGB3 overexpression had the opposite effect of HMGB3 knockdown in SKOV3 cells (*P* < 0.01). Moreover, HMGB3 knockdown and overexpression respectively enhanced and weakened effects of GLA in repressing the malignant characteristics of SKOV3 cells (*P* < 0.05) (**Figures 3B–F** and **Figure 4**).

### GLA Inhibited the Wnt-β-Catenin Signaling Pathway *via* Suppressing HMGB3

Wnt-β-catenin pathway is closely related to EOC development. GLA treatment significantly reduced the expression of HMGB3, Wnt3a, and β-catenin in SKOV3 cells (*P* < 0.01). HMGB3 knockdown had the same effect as GLA treatment, and HMGB3 overexpression exhibited the opposite effect (**Figure 4**). Meanwhile, HMGB3 overexpression weakened effects of GLA by decreasing the protein expression of Wnt3a and β-catenin (*P* < 0.05) (**Figure 4**).

### MiR-374b-5p Directly Targeted HMGB3 in EOC Cells

**Figure 5:**
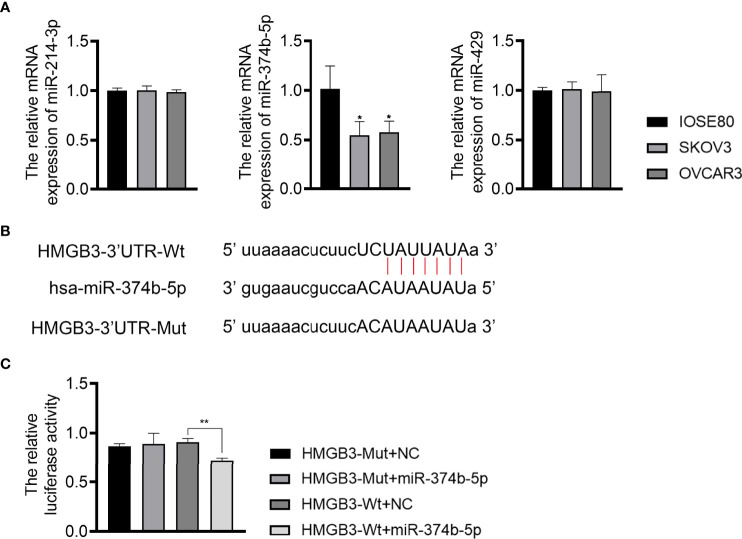
MiR-374b-5p was predicted to directly target HMGB3. **(A)** The relative expression of miR-374b-5p, miR-429, and miR-214-3p in EOC cell lines (SKOV3 and OVCAR3) and those of normal IOSE80 cells was detected by qPCR. ^*^ *P* < 0.05 vs. IOSE80. **(B)** The binding site of has-miR-374b-5p and HMGB3. **(C)** The interaction between miR-374b-5p and HMGB3 was validated through dual-luciferase reporter assay. ^**^ *P* < 0.01.

To understand the upstream mechanisms of HMGB3 in EOC, bioinformatics analysis was performed to predict potential miRNAs targeting HMGB3 using the ENCORI online tool. Results found that miR-374b-5p, miR-429, and miR-214-3p had presumptive binding sites with HMGB3. qPCR revealed that miR-374b-5p expression was dramatically downregulated in SKOV3 and OVCAR3 cells compared to that in IOSE80 cells (*P* < 0.05) (**Figure 5A**). However, the levels of miR-429 and miR-214-3p were not significantly different among IOSE80, SKOV3, and OVCAR3 cells (**Figure 5A**). As presented in **Figure 5B**, the sequence of has-miR-374b-5p has the binding site with the 3’UTR of HMGB3-Wt. The interaction between miR-374b-5p and HMGB3 was verified using a dual-luciferase reporter assay. Upregulation of miR-374b-5p markedly suppressed luciferase activity in SKOV3 cells transfected with HMGB3-Wt (*P* < 0.01); however, this effect was not observed in cells transfected with HMGB3-Mut (**Figure 5C**).

### GLA Suppressed the Malignant Characteristics of EOC Cells by Affecting the MiR-374b-5p/HMGB3 Axis

**Figure 6:**
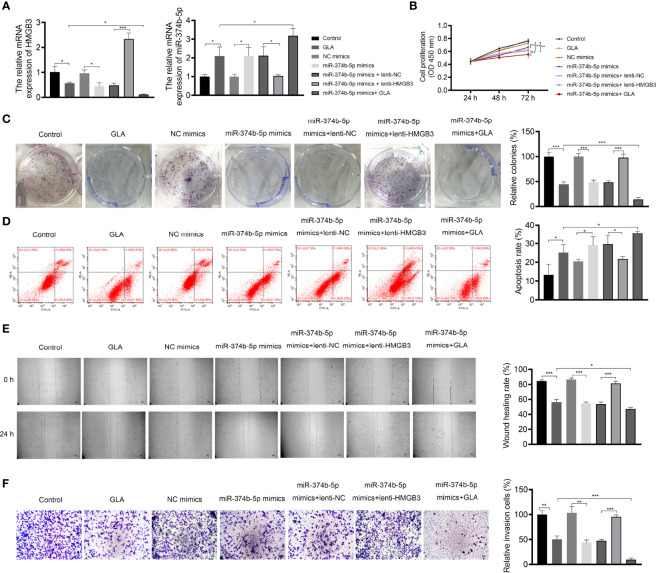
GLA inhibited the malignant characteristics of EOC cells by affecting miR-374b-5p/HMGB3 axis. **(A)** The relative mRNA expression of miR-374b-5p and HMGB3 in SKOV3 cells was measured by qPCR. **(B)** The viability of SKOV3 cells was detected by CCK-8 assay. **(C)** The proliferation of SKOV3 cells was detected by colony formation assay. **(D)** The apoptosis of SKOV3 cells was detected by flow cytometry. **(E)** The migration of SKOV3 cells was detected by wound healing assay. **(F)** The invasion of SKOV3 cells was detected by the Transwell assay. Scale bar = 50 µm. SKOV3 cells were treated with GLA, miR-374b-5p mimics/NC mimics, and/or lenti-HMGB3/lenti-NC. ^*^ *P* < 0.05, ^**^ *P* < 0.01, ^***^ *P* < 0.001.

**Figure 7:**
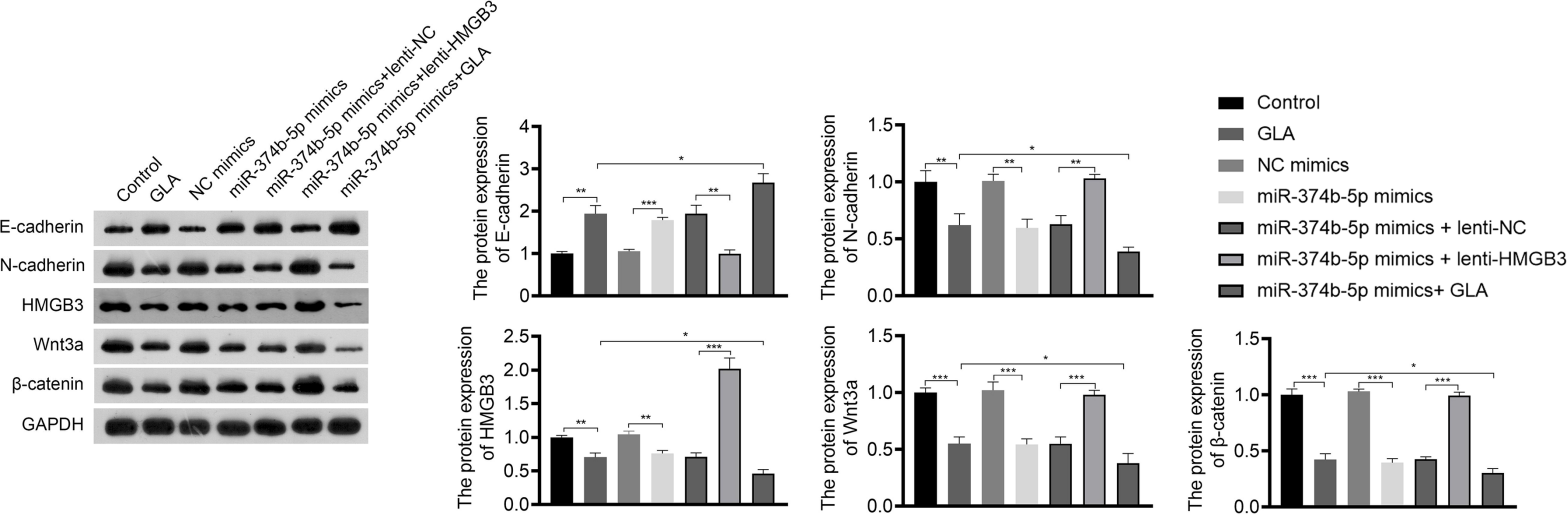
GLA inhibited the expression of EMT and Wnt-β-catenin pathway-related proteins *via* regulating miR-374b-5p/HMGB3 axis. The relative protein expression of E-cadherin, N-cadherin, HMGB3, Wnt3a, and β-catenin in SKOV3 cells in SKOV3 cells was detected by western blotting. SKOV3 cells were treated with GLA, miR-374b-5p mimics/NC mimics, and/or lenti-HMGB3/lenti-NC. ^*^ *P* < 0.05, ^**^ *P* < 0.01, ^***^ *P* < 0.001.

The mechanisms of action of GLA, involving the miR-374b-5p/HMGB3 axis, were further explored. As exhibited in **Figure 6A**, GLA treatment or/and miR-374b-5p overexpression increased the expression of miR-374b-5p and decreased the HMGB3 expression, whereas HMGB3 elevation reversed the effects of miR-374b-5p overexpression (*P* < 0.05). miR-374b-5p overexpression significantly inhibited SKOV3 cell proliferation and colony formation and promoted the apoptosis compared to the those of NC mimics (*P* < 0.05) (**Figures 6B-D**). Meanwhile, miR-374b-5p overexpression downregulated the Bcl-2 level and upregulated the Bax level in SKOV3 cells (*P* < 0.01). Lenti-HMGB3 offset the pro-apoptotic effect of miR-374b-5p, whereas GLA enhanced the effect of miR-374b-5p (*P* < 0.01) (**Supplementary Figure 2**). The migration and invasion of SKOV3 cells were reduced by miR-374b-5p overexpression (*P* < 0.01) (**Figures 6E, F**). MiR-374b-5p overexpression also increased E-cadherin and decreased N-cadherin levels in SKOV3 cells compared to those of the NC mimics (*P* < 0.01) (**Figure 7**). In addition, miR-374b-5p overexpression further enhanced the repressive effects of GLA in SKOV3 cells. Moreover, HMGB3 overexpression partially offset anti-tumor effects of miR-374b-5p overexpression (*P* < 0.01) (**Figures 6B–F** and **Figure 7**).

### GLA Inhibited the Wnt-β-Catenin Pathway by Activating the MiR-374b-5p/HMGB3 Axis

The regulatory relationship between miR-374b-5p/HMGB3 and the Wnt-β-catenin pathway was further investigated. As shown in **Figure 7**, overexpression of miR-374b-5p significantly downregulated the expression of HMGB3, Wnt3a, and β-catenin in SKOV3 cells compared to those in the NC mimics (*P* < 0.05). MiR-374b-5p overexpression enhanced the effect of GLA on inhibiting the Wnt-β-catenin pathway (*P* < 0.05). Besides, HMGB3 overexpression reversed the negative effects of miR-374b-5p overexpression on the expression of Wnt3a and β-catenin in SKOV3 cells (*P* < 0.05) (**Figure 7**).

### GLA Restrained the Tumor Growth of EOC *via* Regulating the MiR-374b-5p/HMGB3/Wnt-β-Catenin Pathway Axis *In Vivo*

**Figure 8:**
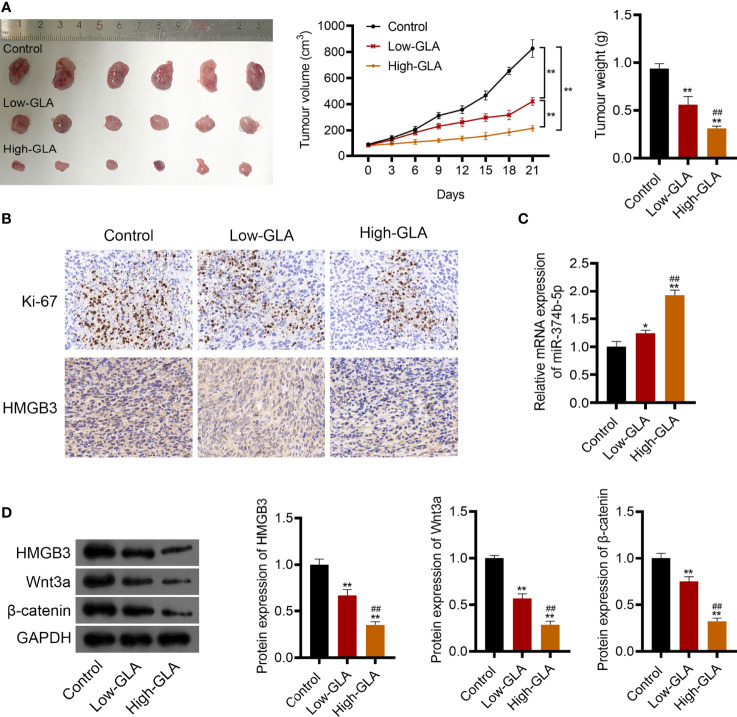
GLA restrained the EOC tumor growth *via* affecting miR-374b-5p/HMGB3/Wnt-β-catenin pathway axis. BALB/c nude mice were subcutaneously injected SKOV3 cells and treated with low- or high-dose GLA. **(A)** Tumor volume and weight were recorded. **(B)** The expression of Ki-67 (a cell proliferation marker) and HMGB3 in tumor tissues was identified by immunohistochemistry. Scale bar = 20 µm. **(C)** The expression of miR-374b-5p in tumor tissues. **(D)** The protein levels of HMGB3, Wnt3a, and β-catenin in tumor tissues. *P <0.05 and **P <0.01 vs. control; ##P <0.01 vs. the low- GLA.

To explore the efficacy and mechanism of GLA on EOC *in vivo*, mice were subcutaneously injected with SKOV3 cell suspension and then with low- or high-dose GLA. As illustrated in **Figure 8A**, after low- or high-dose GLA treatment, the tumor tissues isolated from nude mice presented the decreased size, volume, and weight (*P* < 0.01). Ki-67 (a proliferation marker)-positive cells were also reduced in EOC tumor tissues after GLA treatment (**Figure 8B**). Consistent with cell experiments, miR-374b-5p expression was upregulated in EOC tumor tissues by GLA treatment, whereas the levels of HMGB3, Wnt3a, and β-catenin were downregulated (*P* < 0.05) (**Figures 8B–D**). Further, the role of HMGB3 in EOC tumor was further confirmed. *In vivo* experiments showed that HMGB3 knockdown significantly reduced tumor volume than that in the control (*P* < 0.01) (**Supplementary Figures 3A, B**). In addition to HMGB3-positive cells, Ki-67-positive cells were also decreased in HMGB3 knockdown tumor tissues (**Supplementary Figure 3C**).

## Discussion

EOC is a lethal gynecological malignancy with limited therapeutic options, and Chinese medicines have recently become promising agents for EOC treatment (27). GLA is an ent-kaurene diterpenoid extracted from the Chinese herb *Rabdosia japonica* var with antitumor activity (28). Considerable evidence has verified that GLA is a potential therapeutic drug for cancer treatment (6–8, 29). In this study, we found that GLA treatment dose-dependently reduced EMT and proliferation of EOC cells, suggesting that GLA may inhibit the metastasis and growth of EOC.

HMGB3, from the high-mobility group box subfamily, participates in the cell proliferation, metastasis, and apoptosis in cancers (18, 22). Consistent with results of the previous studies (20, 30, 31), we found that HMGB3 was upregulated in EOC tissues and cells. *In vivo* experiments further confirmed that HMGB3 knockdown diminished the tumor volume and reduced the protein level of Ki-67 in mice. These results indicate that HMGB3 is an oncogene that contributes to EOC development. Moreover, we found that the protein level of HMGB3 was decreased by GLA treatment in EOC cells. Combined with the antitumor effect of GLA, we suspected that GLA may inhibit EOC development, probably by suppressing HMGB3. Our subsequent validation assays determined that HMGB3 overexpression weakened effects of GLA on the inhibition of EOC cell proliferation, migration, invasion, and EMT. These discoveries demonstrated that GLA inhibited the malignant progression of EOC by downregulating HMGB3 expression.

The Wnt-β-catenin signaling pathway is closely involved in regulating pivotal cellular functions, including proliferation, differentiation, migration, and apoptosis (32). HMGB3 promotes tumor development by moderating the Wnt-β-catenin pathway (19, 20, 33). Our results showed that HMGB3 knockdown decreased the protein levels of Wnt3a and β-catenin in EOC cells. This result confirmed that the downregulation of HMGB3 inhibits the Wnt-β-catenin pathway in EOC cells. GLA also inhibits the Wnt-β-catenin pathway, whereas HMGB3 overexpression reversed inhibitory effects of GLA on the protein expression of Wnt3a and β-catenin. Therefore, we considered that GLA exerts an anti-tumor effect in EOC by suppressing the HMGB3-regulated Wnt-β-catenin pathway.

miRNAs play key regulatory roles in cell proliferation, differentiation, and apoptosis during EOC development by regulating their target genes (34). Our present study found that HMGB3 was a downstream target of miR-374b-5p. MiR-374b-5p can affect the progression of various cancers. Zhao et al. have showed that miR-374b-5p suppresses EMT and tumor growth in pancreatic cancer (35). Li et al. demonstrated that miR-374b-5p acts as the tumor suppressor and prognostic biomarker in non-small cell lung cancer (36). In addition, miR-374b-5p abundance is decreased in tumor tissues of ovarian cancer, and its restoration suppresses cell proliferation, migration, and EMT (37). Consistent with previous findings, our study found that miR-374b-5p overexpression restrained proliferation, invasion, migration, and EMT and facilitated the apoptosis of EOC cells. These results illustrate the antitumor effect of miR-374b-5p in EOC. In addition, HMGB3 overexpression partially reversed repressive effects of miR-374b-5p on SKOV3 cell proliferation. Combined with the direct action of GLA on HMGB3, we conclude that GLA suppresses EOC progression by modulating the miR-374b-5p/HMGB3 axis. Moreover, miR-374b-5p overexpression also retarded the Wnt-β-catenin pathway, and this phenomenon was eliminated by HMGB3 overexpression. These results confirm the role of the regulatory axis of the miR-374b-5p/HMGB3/Wnt-β-catenin pathway in EOC. *In vivo* experiments further validated that GLA inhibits EOC tumor growth *via* regulating the miR-374b-5p/HMGB3/Wnt-β-catenin pathway axis.

In conclusion, GLA inhibited the cell proliferation, migration, invasion, EMT, and tumor growth of EOC *in vitro* and *in vivo*. The anti-tumor effect of GLA on EOC is closely related to the miR-374b-5p/HMGB3/Wnt-β-catenin pathway axis. These findings provide a promising drug for EOC treatment and clarify the underlying mechanism of action of GLA in EOC progression. However, the therapeutic effect of GLA on EOC is needed to be further validated clinically. In addition, the mechanism of action of GLA in EOC is not limited to this axis. Further research on the therapeutic mechanisms of GLA in EOC is therefore required.

## Data Availability Statement

The original contributions presented in the study are included in the article/**Supplementary Materials**. Further inquiries can be directed to the corresponding author.

## Ethics Statement

The studies involving human participants were reviewed and approved by the Ethics Committee of Xuzhou Central Hospital. The ethics committee waived the requirement of written informed consent for participation. The animal study was reviewed and approved by the Ethics Committee of Xuzhou Central Hospital. Written informed consent was not obtained from the individual(s) for the publication of any potentially identifiable images or data included in this article.

## Author Contributions

Study design, FC and BZ. Data collection, FC, FS, and XL. Analysis and interpretation, FC, XL, and JS. Statistical analysis, FS, XL, and JS. Drafting manuscript, FC. Revision manuscript, FC and BZ. All authors contributed to manuscript revision, read, and approved the submitted version.

## Funding

This work was supported by Jiangsu traditional Chinese medicine science and technology development plan project [project no. ZT202114].

## Conflict of Interest

The authors declare that the research was conducted in the absence of any commercial or financial relationships that could be construed as a potential conflict of interest.

## Publisher’s Note

All claims expressed in this article are solely those of the authors and do not necessarily represent those of their affiliated organizations, or those of the publisher, the editors and the reviewers. Any product that may be evaluated in this article, or claim that may be made by its manufacturer, is not guaranteed or endorsed by the publisher.

## Supplementary Material

Supplementary Figure 1 GLA inhibited the apoptosis of EOC cells by regulating HMGB3. Relative protein expression of Bcl-2 and Bax in SKOV3 cells was measured by western blotting. ^**^ *P* < 0.01.

Supplementary Figure 2 GLA inhibited the apoptosis of EOC cells by affecting miR-374b-5p/HMGB3 axis. Relative protein expression of Bcl-2 and Bax in SKOV3 cells was measured by western blotting. ^**^ *P* < 0.01.

Supplementary Figure 3 HMGB3 silencing suppressed the tumor growth of EOC *in vivo*. **(A)** SKOV3 cells transfected with sh-NC or sh-HMGB3 were injected subcutaneously into nude mice, and tumor growth was observed. **(B)** Tumor growth curves were recorded by measuring tumor volume. ^**^ *P* < 0.01. **(C)** The expression of HMGB3 and Ki-67 in tumor tissues was identified by immunohistochemistry. Scale bar = 20 µm.
