# Supplementary figures and images for: Glaucocalyxin A Inhibits the Malignant Progression of Epithelial Ovarian Cancer by Affecting the MicroRNA-374b-5p/HMGB3/Wnt-β-Catenin Pathway Axis
Source: Front Oncol. 2022 Jul 14;12:955830. doi: 10.3389/fonc.2022.955830 (PMC9329791; doi:10.3389/fonc.2022.955830)

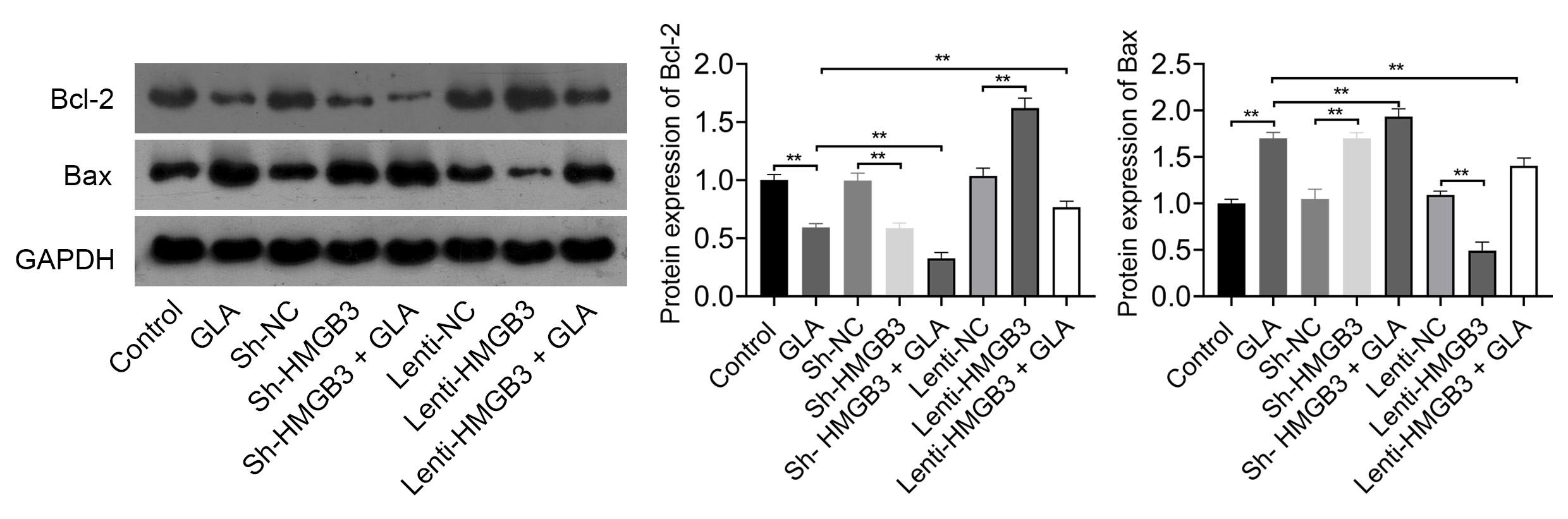

Supplement: Supplementary Figure 1 — GLA inhibited the apoptosis of EOC cells by regulating HMGB3. Relative protein expression of Bcl-2 and Bax in SKOV3 cells was measured by western blotting. ** P < 0.01. [file DataSheet_1.zip › Supplementary file/Supplementary Figure 1.tif]

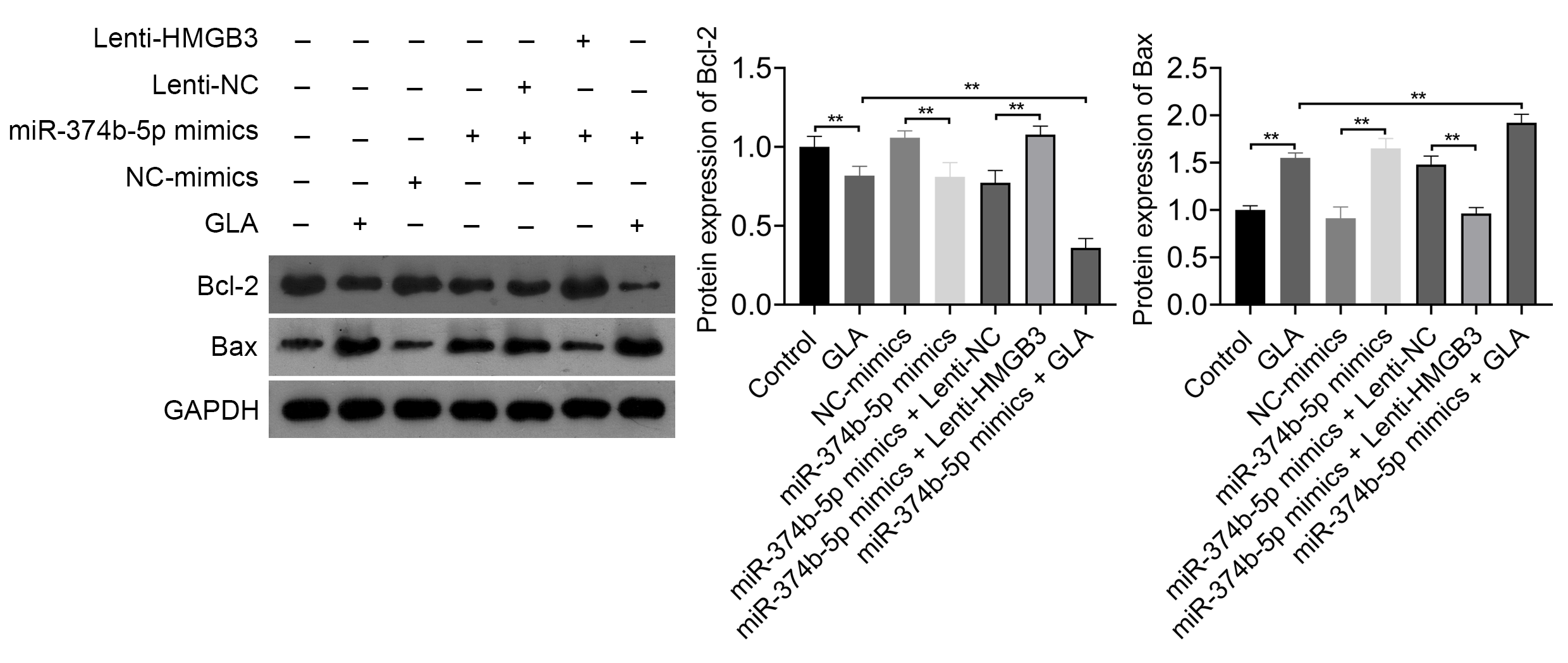

Supplement: Supplementary Figure 1 — GLA inhibited the apoptosis of EOC cells by regulating HMGB3. Relative protein expression of Bcl-2 and Bax in SKOV3 cells was measured by western blotting. ** P < 0.01. [file DataSheet_1.zip › Supplementary file/Supplementary Figure 2.tif]
