# Supplementary material for: Glaucocalyxin A Inhibits the Malignant Progression of Epithelial Ovarian Cancer by Affecting the MicroRNA-374b-5p/HMGB3/Wnt-β-Catenin Pathway Axis
Source: Front Oncol. 2022 Jul 14;12:955830. doi: 10.3389/fonc.2022.955830 (PMC9329791; doi:10.3389/fonc.2022.955830)
Supplement: Supplementary file 2 [file Table_1.docx]

The raw data is uploaded to a public database with a link to https://www.jianguoyun.com/p/DSJPR8sQoNjPChjcoMcEIAA
